# A factorial cluster-randomised controlled trial combining home-environmental and early child development interventions to improve child health and development: rationale, trial design and baseline findings

**DOI:** 10.1186/s12874-020-00950-y

**Published:** 2020-04-02

**Authors:** Stella M. Hartinger, Nestor Nuño, Jan Hattendorf, Hector Verastegui, Walter Karlen, Mariela Ortiz, Daniel Mäusezahl

**Affiliations:** 1 Department of Epidemiology & Public Health, Swiss Tropical and Public Health Institute, Basel, Switzerland; 2 School of Public Health and Administration, Universidad Peruana Cayetano Heredia, Lima, Peru; 3 University of Basel, Basel, Switzerland; 4 Department of Health Sciences and Technology, ETH Zürich, Zürich, Switzerland; 5 Programa Nacional CUNA MAS, Lima, Peru

**Keywords:** Cluster-randomised trial, Integrated home-based interventions, Early child development, Diarrhoea, Respiratory infections, Kitchen hygiene, Household air pollution, Household water treatment, Improved biomass cookstoves, Peru

## Abstract

**Background:**

Exposure to unhealthy environments and inadequate child stimulation are main risk factors that affect children’s health and wellbeing in low- and middle-income countries. Interventions that simultaneously address several risk factors at the household level have great potential to reduce these negative effects. We present the design and baseline findings of a cluster-randomised controlled trial to evaluate the impact of an integrated home-environmental intervention package and an early child development programme to improve diarrhoea, acute respiratory infections and childhood developmental outcomes in children under 36 months of age living in resource-limited rural Andean Peru.

**Methods:**

We collected baseline data on children’s developmental performance, health status and demography as well as microbial contamination in drinking water. In a sub-sample of households, we measured indoor kitchen 24-h air concentration levels of carbon monoxide (CO) and fine particulate matter (PM_2.5_) and CO for personal exposure.

**Results:**

We recruited and randomised 317 children from 40 community-clusters to four study arms. At baseline, all arms had similar health and demographic characteristics, and the developmental status of children was comparable between arms. The analysis revealed that more than 25% of mothers completed primary education, a large proportion of children were stunted and diarrhoea prevalence was above 18%. Fifty-two percent of drinking water samples tested positive for thermo-tolerant coliforms and the occurrence of *E.coli* was evenly distributed between arms. The mean levels of kitchen PM_2.5_ and CO concentrations were 213 μg/m^3^ and 4.8 ppm, respectively.

**Conclusions:**

The trial arms are balanced with respect to most baseline characteristics, such as household air and water pollution, and child development. These results ensure the possible estimation of the trial effectiveness. This trial will yield valuable information for assessing synergic, rational and cost-effective benefits of the combination of home-based interventions.

**Trial Registry:**

ISRCTN-26548981.

## Background

Children in low- and middle-income countries are frequently exposed to cumulative health and developmental risks often rooted in unhealthy environments [1], with household air pollution (HAP) and water, sanitation and hygiene (WASH) being the main risk factors [2]. Unfortunately, 40% of the world population still relies on solid fuels for cooking [3], 11% does not have access to an unimproved drinking water source and 36% lacks sanitation [4]. These risk factors disproportionally target the poor and vulnerable increasing the burden of communicable (acute respiratory infections [5], pneumonia [6], diarrhoea [7, 8]) and non-communicable diseases (COPD [9], adverse birth outcomes [10], malnutrition [11, 12]) in these populations. Overall, HAP accounts for 3.8 million deaths [13] and WASH for 0.8 million deaths worldwide [12]. Child developmental outcomes such as cognitive deficits, neurodevelopment affectation [14, 15], sensorimotor and socio-emotional development [16] have been linked to unhealthy environments, especially if exposure occurs during the first years of life [17, 18]. Early child development (ECD) generates opportunities that shape children’s lifelong health and developmental status [18]; however, around two hundred million children worldwide are not reaching their full cognitive potential [14], increasing the in equity between populations, and promoting lifetime adverse consequences that impact the wellbeing of future generations [15, 19]. Hence, improving access to basic services through structural household improvements such as latrines, running water in-house or ventilation-improved cooking devices on the one hand, as well as improving life course determinants at an early age on the other hand are promising interventions to reduce the household burden of disease.

Individual WASH and HAP interventions reduce the burden of disease. A recent systematic review showed that point-of-use filtration and water disinfection interventions reduced the risk of diarrhoea by 53 and 31% respectively; hygiene education focusing on handwashing with soap resulted in a 27% reduction [7]. In addition, continuous availability of higher quality water piped to households can also reduce diarrhoea by 75% [20]. Improved cookstoves (ICS) interventions have also shown impact on HAP exposure reduction [21], improved health [22, 23] and wellbeing. ECD interventions enhanced health status [24] and development in children [25].

The improvement of poor health and development requires integrated approaches addressing the underlying social, behavioural risks and household infrastructural determinants at the household level [16, 26]. Multiple studies have demonstrated that low-cost interventions focusing on various health risk factors simultaneously had a synergic effect and increased opportunities for sustained use of them over time [27, 28]. Integrating ECD and home-hygiene interventions is promising as they both target young children within their homes [29, 30]. The home-based approach enhances the effects of ECD interventions, which could also lead to improvements of children’s general health and affection and mothers’ mental health [31, 32]. Furthermore, this combination can improve the effectiveness of each intervention alone and reduce the use of material and human resources compared to the implementation of these interventions independently. In this article, we describe the design and baseline results of a cluster-randomised controlled trial seeking to develop an integrated cost-effective home-based package (IHIP) of environmental health and ECD interventions in rural Andean Peru.

## Methods

### Setting and subjects

The trial was conducted in the San Marcos and Cajabamba provinces, Cajamarca region, Andean Peru. We chose the locations based on the coverage of the national ECD programme (“Programa Nacional Cuna Mas” (PNCM)) and on-going relationships with local stakeholders who partook in a previous cluster-randomised controlled trial conducted in the region. The results of our previous research endeavours facilitated the implementation of this new trial (“IHIP-2 trial” in the following) [25, 29]. Both sites were high altitude rural resource-limited locations with chronic malnutrition and illiteracy [33]. The majority of the population were small-scale farmers living in 2–3 roomed houses with earthen floors and adobe walls, with traditional stoves or open fires for cooking.

### Study design

We implemented a 2 × 2 factorial design trial applying two interventions individually and in combination: i) an environmental health package comprising a certified ICS, kitchen sink and hygiene education (IHIP); and ii) an early child development programme (ECD). This design led to four potential experimental conditions: i) IHIP & ECD (“IHIP+” in the following), ii) IHIP, iii) ECD and iv) Control. We chose this design because it randomises communities instead of individuals, avoiding contamination between participants.

We enrolled all families complying with the following inclusion criteria: i) had at least one child < 1.5 years living at the household; ii) used solid fuels as main energy source for cooking/heating; iii) had access to piped water in the yard; iv) did not plan to move within the next 24 months; and iv) did not participate in the PNCM (Additional file 1).

### Sample size

We calculated the sample size using the formula proposed by Hayes & Bennett for cluster-randomised trials [34], assuming three episodes of diarrhoea per child-year, a 25% reduction of the incidence compared to the control arm, and a coefficient of variation of 0.2. With 10 person-years of follow-up in each cluster, we calculated 16 clusters for the intervention and control arm to detect the anticipated reduction of incidence with a power of 80% at the 5% two-sided significance level. To account for potential loss to follow-up, we included 40 clusters with an average of 6.5 person-years of follow-up. For the ECD intervention, we used the ECD outcome (percentage of tasks solved above the mean of the study population) of our previous intervention study [25] and assumed 60% above mean for the intervention and 40% above mean in the control arm. Using the equivalent formula for proportions, we calculated that 15 clusters for intervention and control with ten children were required. Of note, the trial is sufficiently powered to compare each intervention against the corresponding control arms (i.e. for IHIP arm 1&2 vs 3&4 and for ECD arm 1&3 vs 2&4) but does not allow pair wise comparisons among the four trial arms.

### Recruitment

We carried out a census in 2015 to identify potential communities, children and pregnant women in their second and third trimester in collaboration with the Peruvian Ministry of Health (MINSA). Participants were enrolled between September 2015 and January 2016. If multiple eligible children were found in a household, we selected the youngest child.

### Randomisation

We enrolled 82 eligible communities, which we aggregated into 40 community-clusters because of their partly close proximity to each other. After the enrolment, we allocated the communities into the four study arms using a covariate-based constrained randomisation as proposed by Moulton [35]. Based on geographic distance, we first divided the clusters into 8 strata of 4 clusters each and 1 stratum of 8 clusters. We then generated two million random allocation sequences and selected those for which the maximum difference between arms was i) ≤ 5 in terms of number of villages; ii) ≤10 for the number of children; iii) median village size (number of households) ≤10; iv) median altitude ≤250; v) proportion of households with a health post in the community ≤10%-points; vi) proportion of households with a school within the community ≤10%-points; and vii) proportion of households living in villages having an electricity connection ≤10%-points. Of the 164 allocation sequences that fulfilled all criteria, one was randomly selected.

### Design and implementation of study interventions

#### The IHIP intervention

The IHIP package was developed to improve unsafe drinking water, hygienic conditions and HAP levels and, consequently, enhance potential effects to reduce diarrhoea and respiratory infections. It was designed using an extended community participatory approach on the needs and preferences of rural Andean populations [28, 36]. To select the ICS model, we consulted communities on their preference regarding three pre-selected ICS between May and July 2015. Forty-eight women were asked to cook with each ICS model for three consecutive days (9 in total). Participant women selected the ICS “OPTIMA” model and recommended further modifications before its installation. The modified stove was certified by the Peruvian national industrial certification authority (SENCICO) [37]. To increase availability of water inside the household and convenience in accomplishing household chores (both were expressed needs and voiced at user consultations), prefabricated cement sinks were installed inside the kitchen and connected to the local water supply found in the household yard (between 5 and 20 m away from the kitchen).

Kitchen sinks and stove parts were purchased locally to increase scalability. Both interventions were implemented between November 2015 and February 2016. Activities of the hygiene education component were carried out continuously (every month) throughout the study; this was done with the aid of a small flip chart. We conveyed three main messages: i) Kitchen hygiene: elimination of animal excreta and keeping the kitchen environment clean; ii) Hand washing: of the mother’s and child’s hands with soap or detergent at key moments during the day (after using the toilets, changing diapers, before preparing the meals and before eating); and iii) household water treatment. We especially promoted boiling, since it is the main HWT endorsed by the health authorities in the area. All other HWT methods were also discussed during the sessions.

#### Early child development intervention

The ECD intervention was based on the PNCM [38]. The PNCM was launched in 2012 to improve the cognitive, social and emotional development of infants < 3 years of age living in poverty. In addition, the programme sought to improve families’ knowledge and practices regarding caregiving, and strengthen the bond between mothers and children. The PNCM provides a home-visiting intervention in rural areas (“Acompañamiento a Familias” (AAF)) as opposed to a day-care service in urban settings. The AAF was implemented and evaluated in our trial.

Mother facilitators (MF) were trained women living in the participating communities and conducted weekly play-oriented, semi-structured activities with the participant mothers (or caretakers) and the child. MFs were selected using PNCM guidelines and received a one-day training session and monthly re-trainings. MFs were supervised by a technical assistant team (TA), which mentored and assisted MFs in the planning of the weekly sessions according to the PNCM guidelines. TA team organised group sessions with participant mothers every two months to share experiences, lessons or concerns of the ECD intervention. The TA team received a one-week training session from PNCM experts in the planning and delivery of the ECD sessions. All participating households received a set of age-specific toys to stimulate children’s psychomotor and cognitive development every 2 months (six packages in total). These educational materials aimed to foster communicative, socio-emotional and cognitive abilities and to teach mothers how to plays and equip them with ideas for new games for daily interactions with their child.

### Primary and secondary outcomes

#### Definition of the primary and secondary outcomes

We assessed childhood diarrhoea and ECD status as primary outcomes. We defined diarrhoea according to the World Health Organization (WHO) standards, as the passing of at least three loose stools within 24 h [39], and ECD outcomes as an age standardised mean score of psychomotor assessment (including socio-emotional, motor and cognitive skills, and communication abilities) in children < 3 years of age.

The secondary outcomes included: i) acute respiratory infections (ARI), defined as presence of cough and fever reported by the primary caretaker, according to the WHO standards [40]; ii) severe cases of diarrhoea defined as persistent diarrhoea for more than 14 days or bloody diarrhoea; iii) household carbon monoxide (CO) and particle matter (PM_2.5_) emissions and CO personal exposure in a sub-sample of 40 participants; iv) presence of *E.coli* in drinking water samples; and v) compliance linked to the use of the interventions. We assessed compliance with stove and sink use and hygiene through spot check observations, 24-h recall data and direct observations in the sentinel sub-sample. ECD compliance was defined as reported ECD sessions since the last TA’s household visit and mother’s satisfaction associated with the MFs visits.

#### Data collection of primary and secondary outcomes

We carried out active and passive surveillance to collect diarrhoea and ARI morbidity data during the 12-month follow-up. The fieldworker team (FW) visited all households weekly and collected daily and weekly self-reported information from the mother or caretaker about the occurrence of signs and symptoms of child diarrhoea and ARI. The FW was instructed to obtain two measurements of respiratory rates, which increased the specificity for a diagnosis without a loss of sensitivity [41]. To define the severity of the disease, for diarrhoea, we collected additional information on observed blood in the stools. For ARI, we measured respiratory rate, heart rate and oxygen saturation in blood (SpO_2_) with portable pulse oximeters (PPOs) (Masimo iSpO_2_ Rx) and multisite reusable sensors (Masimo M-LNCS YI SpO_2_) connected to tablets (Lenovo Tab 2 A7–10). We used the lambdanative framework [42] to develop a mobile app which provided data entry and real-time feedback on signal quality [43]. Respiratory rate was recorded using the RRate app module [44]. Severely ill children were referred to the local healthcare facility for further evaluation. The FW initially received five-days of training in morbidity data collection with monthly re-training sessions of 2 h. All the surveillance devices and tools were tested on a daily-basis between February and April 2016. In addition, the passive surveillance team (PS) collected health data from our study participants monthly at their local community-based health centres (22 in total). To ensure that they were also collecting SpO_2_ in each evaluation, we provided PPOs, sensors and tablets to local health centres. During the visits, the PS also trained local health personnel on the maintenance and use of the PPOs. Anthropometric measurements were collected from the participant’s clinical records. We assessed stunting and underweight following the WHO standards; stunting: height-for-age z-score < − 2; underweight: weight-for-age z-score < − 2) [45].

The environmental team (EV) collected household air pollution and drinking water samples. The EV received 7 days of initial training and monthly re-training sessions. We collected 24-h kitchen and personal CO and PM_2.5_ exposure data. We installed one EL-USB-CO (LACAR Electronics) monitor and one HAP measuring device for indoor use APROVECHO-5000 at a one-meter distance from the ICS and at standard breathing height (1.5 m). To measure personal exposure, mothers were carrying a vest equipped with one EL-USB-CO (LASCAR Electronics) monitor and one micro personal aerosol exposure monitor (RTI-INTERNATIONAL). We asked mothers to wear the vest for 24 h only taking them off for sleeping and personal hygiene.

We obtained drinking water samples from the child’s main drinking source, and then transported them to the field station’s laboratory in San Marcos, in cooled thermal bags. At the laboratory, the samples were analysed for thermotolerant (faecal) coliforms using a membrane-filtration method from the Oxfam DelAgua water testing kit [46]. All yellow colonies forming units were considered positive for *E.coli* growth. These were then collected and placed in transportation vials media and sent to Lima for further phenotypic and genotypic identification and antibiotic resistance testing.

HAP data were obtained from the sentinel sub-sample five times (before ICS installation, three times during follow-up, and at the end of study). Water samples were collected at baseline and end of study for all study participants and in the sentinel sub-sample three additional times over the follow-up.

To assess ECD status, the TA carried out an assessment using the nationally validated Peruvian Infant Development Scale (ESDI) tool [38] at baseline and end of study. The TA received 1 week of training from PNCM experts. At the end of study, a group of specialized psychologists also conducted the Bayley Scales of Infant and Toddler Development (BSID) [47] instrument for comparability.

Finally, the capacity building team (CBT) was responsible for re-training participants on hygiene, hand-washing, boiling practices and ICS maintenance. The CBT conducted monthly reinforcement visits and collected data on the IHIP intervention condition. A local ICS constructor was hired to supported additional maintenance of the intervention on demand. During their visits, the FW, CBT and TA conducted regular spot check observations and collected maternal reports on the usage and quality of the interventions as well as household and environmental hygiene.

#### Socio-economic survey

The FW implemented a socio-economic questionnaire at baseline and end of study. The objective was to assess household demographics, education and economic characteristics, general stove use and household water management. We conducted the baseline assessment between September 2015 and February 2016. The FW received 1 week of training.

#### Data quality management

The field coordinator team revised information collected on a daily basis to reduce the chance of missing data. They also trained the field staff, double-checked questionnaires and conducted regular household quality visits. Ten percent of all data collected in the trial was double entered to ensure data quality. To reduce the possibility of courtesy bias, household data collection routes of FW were changed every 2 months. The data integrity of physiological data obtained from the PPO and RRate were automatically assessed using validated computer algorithms [44].

#### Data analysis

The data was entered in the Census and Survey Processing System (CS Pro 6.3) and data files were exported to Stata 15 Statistical software (STATA CORP, College Station, Texas, USA) for analysis. We performed a descriptive analysis (means, percentages) for the main household characteristic and for the health characteristics we calculates prevalence and proportions.

## Results

### Enrolment

**Fig. 1:**
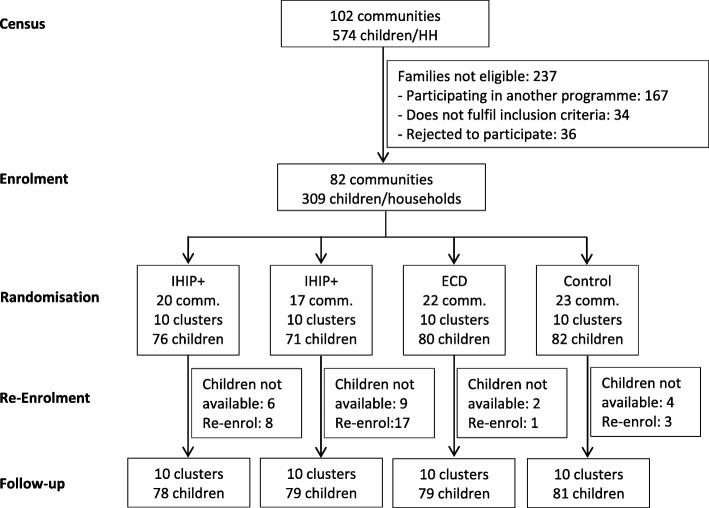
Flow diagram of a cluster-randomised controlled trial in rural Andean Peru

From the screening census, we identified 102 communities with 574 potential children. During enrolment we found that 237 families were no longer eligible because i) they participated in another social programme (*N* = 167); ii) they did not fulfil the inclusion criteria (*N* = 34), or iii) they rejected participation (*N* = 36). We re-enrolled between January and February 2016 because 28 children were not available or rejected to participate in the project at the beginning of the follow-up. In total, 317 households in 10 clusters per arm participated in the trial (Fig. 1).

### Baseline characteristics

Baseline analysis included the demographic and health characteristics, the household drinking water quality and levels of ECD indicators for each participant. We measured household and personal air pollution concentrations in the sentinel sub-sample.

#### Demographic and household characteristics

The four arms were balanced in most of the domains (Table 1). Mothers from the four arms were of similar mean age, had similar levels of primary education and years of schooling. The majority of household had adobe walls, earthen floors, tiled roofs, and piped water system. We observed differences among trial arms in the proportion of children below 1 year. This imbalance disappears comparing IHIP versus no-IHIP or ECD versus no-ECD arms. We also observed imbalance in the proportion of households with electricity. Although living in villages having an electricity connection was a randomisation constraint it did not ensure balance at household level (in villages with connection the proportion of households with electricity varied from 50 to 100%).

**Table 1:** Demographic and household characteristics of households in rural Andean Peru

	IHIP+		IHIP		ECD		Control	
	N	Mean (SD) or % (N)	N	Mean (SD) or % (N)	N	Mean (SD) or % (N)	N	Mean (SD) or % (N)
*Demographic characteristics*	78		79		79		81	
Number of inhabitants per household		4.9 (1.5)		5.1 (1.7)		4.8 (1.4)		5.0 (1.5)
Child sex (female)		43.6% (34)		50.6% (40)		53.2% (42)		55.6% (45)
Child age (years)^a^		1.5 (0.5)		1.5 (0.5)		1.7 (0.5)		1.7 (0.5)
Child < 1 year^a^		20.5% (16)		20.3% (16)		10.1% (8)		9.9% (8)
Age of caretaker (years)		27.3 (7.1)		28.4 (7.4)		27.2 (6.6)		28.0 (6.9)
*Maternal education*	78		79		79		81	
None		6.4% (5)		2.5% (2)		5.1% (4)		7.4% (6)
Primary level completed		30.8% (24)		32.9% (26)		27.9% (22)		23.5% (19)
Secondary level completed		21.8% (17)		6.3% (5)		17.7% (14)		8.6% (7)
Higher degrees completed		0% (0)		3.8% (3)		5.1% (4)		1.2% (1)
Years of schooling^b^		6.1 (3.5)		6.1 (3.3)		6.9 (3.5)		5.6 (3.2)
*Household characteristics*	78		79		79		81	
Adobe wall type		89.7% (70)		92.4% (73)		93.7% (74)		100% (81)
Earthen floor type		83.3% (65)		89.9% (71)		94.9% (75)		93.8% (76)
Roof tile type		82.1% (64)		87.3% (69)		86.1% (68)		95.1% (77)
Household with latrines		55.1% (43)		53.2% (42)		58.2% (46)		61.7% (50)
Piped water supply		97.4% (76)		98.7% (78)		100% (79)		98.8% (80)
Electricity		65.4% (51)		82.3% (65)		77.2% (61)		85.2% (69)

^a^ Age calculated for the start of follow-up (April 1^st^ 2016)

^b^ For higher degrees, we assumed: Non-university education not completed (12.5 years), non-university education completed (14 years), university education not completed (13.5 years) and university education completed (16 years)

#### Health characteristics

Children in all arms had similar weight at birth. Imbalance was observed in the prevalence of stunting, with the highest proportion in the ECD arm (36.9%). The two-week diarrhoea prevalence was balanced between arms, as was the two-week prevalence of cough and fever. However, we did observed differences between the health insurance coverage between arms, with the highest lack of coverage in the IHIP+ (10.3%) and the lowest in the control arm (1.3%). Vaccination coverage, varied between arms (Table 2). The highest complete doses of vaccines for children aged between 6 and 11 months were in the ECD, and the lowest in the control arm (40%). These percentages change when we look at children between aged 12–24 months, where coverage levels in the ECD arm increase up to 70% and the four arms are balanced. SpO_2_ and respiratory rate levels were similar in all arms (Table 2).

**Table 2:** Children’s health status and vaccination coverage in rural Andean Peru

	IHIP+		IHIP		ECD		Control	
	N	Mean (SD) or % (N)	N	Mean (SD) or % (N)	N	Mean (SD) or % (N)	N	Mean (SD) or % (N)
*Anthropometrics*^a^	78		79		79		81	
Height-for-age, Z-values	64	− 1.3 (1.1)	50	−1.0 (0.9)	64	−1.5 (1.1)	61	−1.4 (0.8)
Stunting	64	17.2% (11)	50	9.8% (5)	64	36.9% (24)	61	18.0% (11)
Weight-for-age, Z-scores	64	−0.4 (1.0)	51	− 0.1 (0.8)	65	− 0.6 (0.9)	61	− 0.5 (0.9)
Underweight	64	6.3% (4)	51	2.0% (1)	65	10.8% (7)	61	4.9% (3)
*Children’s health status*	78		79		79		81	
Weight at birth		3.1 (0.6)		3 (0.5)		3.1 (0.5)		3.1 (0.4)
Diarrhoea, two-weeks prevalence		18.4% (14)		22.8% (18)		19% (15)		26.3% (21)
Fever, two-weeks prevalence		21.8% (17)		21.5% (17)		25.3% (20)		23.5% (19)
Cough, two-weeks prevalence		35.1% (27)		31.7% (25)		39.2% (31)		29.1% (23)
Children with no health coverage		10.3% (8)		10.1% (8)		5.1% (4)		1.2% (1)
*Children’s vaccination*	78		79		79		81	
Tuberculosis (BCG)	77	89.6% (69)		91.1% (72)		94.9% (75)		95.1% (77)
Measles	77	50.7% (39)	78	60.3% (47)	77	67.5% (52)		64.2% (52)
Diphtheria, convulsive cough and tetanus (DTP)^b^								
1st dose	77	96.1% (74)		94.9% (75)		98.7% (78)		95.1% (77)
2nd dose	73	90.4% (66)	77	89.6% (69)		97.5% (77)	79	93.7% (74)
3rd dose	64	87.5% (56)	69	79.7% (55)	72	93.1% (67)	73	82.2% (60)
Polio (OPV)^c^								
1st dose	77	96.1% (74)		96.2% (76)		98.7% (78)		96.3% (78)
2nd dose	73	89.0% (65)	77	90.9% (70)		97.5% (77)	79	93.7% (74)
3rd dose	64	78.1% (50)	69	79.7% (55)	72	90.3% (65)	73	75.3% (55)
Influenza	77	62.3% (48)		63.3% (50)		81.0% (64)		69.1% (56)
Hepatitis B	77	62.3% (48)		54.4% (43)		79.8% (63)		69.1% (56)
*Recommended vaccines* 6–11 months	23		28		24		15	
All vaccines^d^		69.6% (16)		60.7% (17)		87.5% (21)		40% (6)
No vaccines		0% (0)		7.1% (2)		0% (0)		0% (0)
*Recommended vaccines* 12–24 months	41		41		48		58	
All vaccines^e^		68.3% (28)		70.7% (29)	46	84.8% (39)		70.7% (41)
No vaccines		2.4% (1)		4.9% (2)		2.1% (1)		0% (0)
*Portable pulse oximetry assessment*^f^	64		59		63		68	
Oxygen saturation (SpO_2_)		93.5 (2.8)		94.2 (2.5)		93.8 (3.8)		93.8 (2.8)
Respiratory rate per/min.		27.7 (3.7)		26.4 (3.0)		27.2 (2.7)		27.3 (3.1)

^1^As estimated on 15^th^ December 2015 (±31 days). If several estimates within the range were available, the one closest to 15^th^ of December was selected

^b^DPT vaccination: 1^st^ dose: 2 month; 2^nd^ dose: 4 month; and 3^rd^ dose: 6 month

^c^ Polio vaccination: 1^st^ dose: 2 month; 2^nd^ dose: 4 month; and 3^rd^ dose: 6 month

^d^ It includes BCG (one dose), DTP (three doses) and OPV (three doses)

^e^ It includes BCG (one dose), DTP (three doses), OPV (three doses) and measles (one dose)

^f^ Results from children with no observable danger signs, fever, cough and difficulty to breath at the time of the assessment

### Household microbial contamination

We obtained 314 water samples. Some 52.9% (*N* = 166) tested positive for thermo-tolerant bacteria. The number of positives and the occurrence of *E.coli* and *Enterobacter* were evenly distributed between arms. We observed imbalances in the occurrence of *Klebsiella* and *Citrobacter*, although the number of cases were small (Table 3).

**Table 3:** Thermo-tolerant bacteria from household drinking water samples in rural Andean Peru

	IHIP+		IHIP		ECD		Control		Total	
	N	% (N)	N	% (N)	N	% (N)	N	% (N)	N	% (N)
	38		37		43		48		166	
*E.coli*		71.1% (27)		73.0% (27)		65.1% (28)		72.9% (35)		70.5% (117)
*Klebsiella*		10.5% (4)		8.1% (3)		20.9% (9)		18.8 (9)		15.1% (25)
*Enterobacter*		10.5% (4)		10.8% (4)		9.3% (4)		8.3% (4)		9.7% (16)
*Citrobacter*		7.9% (3)		8.1% (3)		4.7% (2)		0% (0)		4.8% (8)

### Household and personal air pollution

We measured indoor PM_2.5_ and CO concentrations stationary from the kitchen environments and personal exposure (CO only) before the installation of the ICS. Data was collected from 33 households. The average household PM_2.5_ and CO concentration were 213 μg/m^3^ (SD = 166.1) and 4.8 ppm (SD = 3.7) respectively (Table 4).

**Table 4:** HAP measurements at baseline in rural Andean Peru

	N	Mean (95% IC)	Median (95% IC)	Geometric mean (95% IC)
*Kitchen*	33			
PM_2.5_ (μg/m^3^)		213 (154.9–271.2)	46.5 (23.5–64.6)	48.1 (37.5–61.7)
CO (ppm)		4.8 (3.5–6.2)	1.1 (0.7–1.7)	1.3 (1.0–1.7)
*Personal*	33			
CO (ppm)		1.4 (0.8–2.0)	0 (0–0)	2.4 (1.9–3.1)

### Early child development assessment

**Fig. 2:**
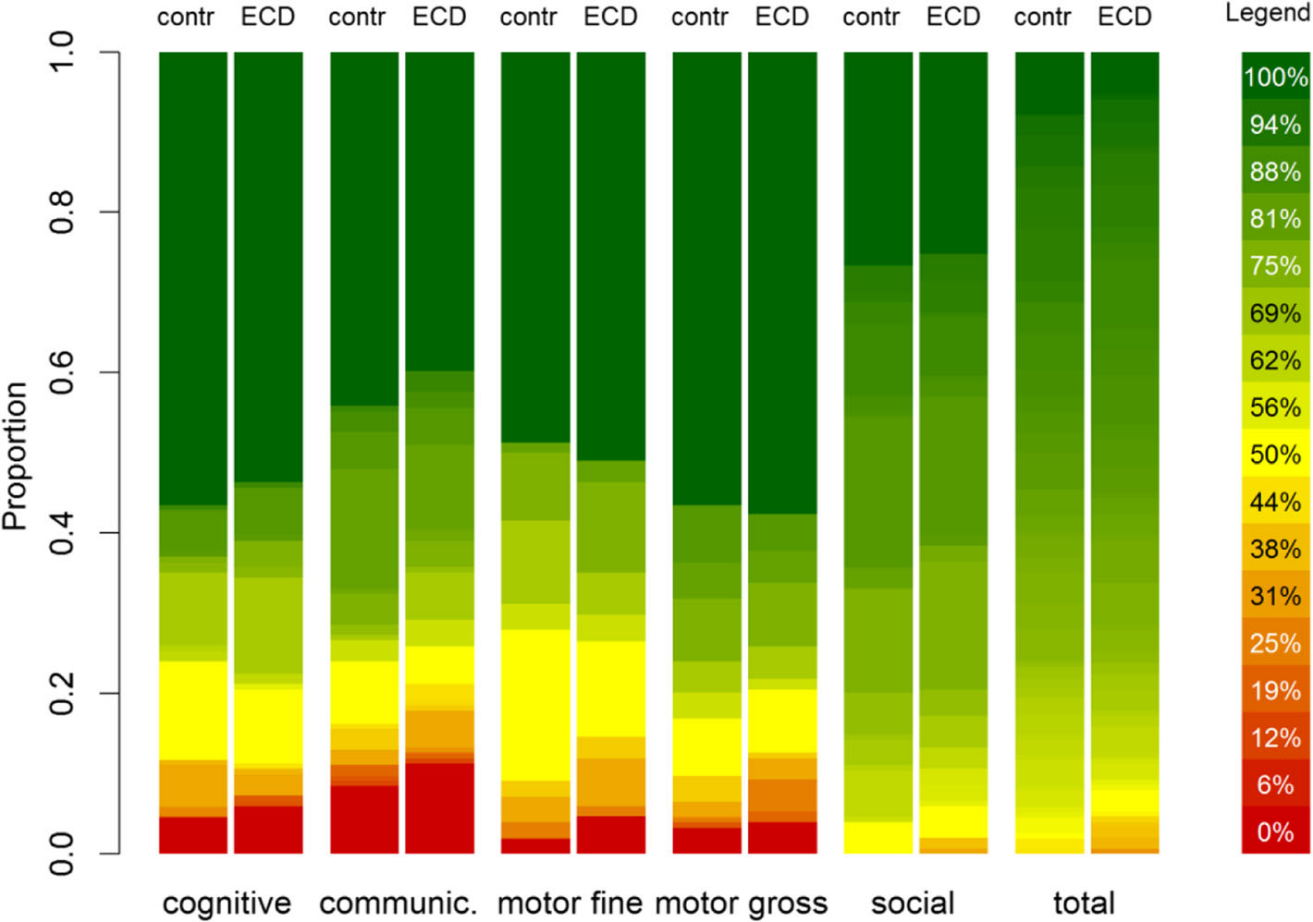
Proportion of tasks completed for all early child developmental domains

Child psychomotor and cognitive development indicators of 305 study children indicated similar performance in all developmental domains across arms (Fig. 2).

## Discussion

We present the baseline results of a cluster-randomised trial evaluating an integrated home-environmental intervention package and an ECD programme to improve diarrhoea, ARI and developmental outcomes in children under 36 months of age living in resource-limited rural Andean Peru. This trial uses a robust 2 × 2 factorial design that allows the assessment of two interventions in a single study without increasing sample size [48]. For the ECD intervention, we implemented one component of Peru’s national ECD programme (PNCM).

The trial included 317 rural families living in 82 communities from two Peruvian provinces. Baseline results indicate the trial arms are balanced with respect to most baseline characteristics, but given the limited number of clusters and the amount of characteristics presented, we also found a few imbalances. Imbalances observed will be considered in the primary trial analysis comparing each intervention separately with its counterfactual, i.e. IHIP versus no-IHIP to assess the impact of the IHIP intervention, and ECD versus no-ECD arms to evaluate the effects of the ECD intervention. While the ECD performance measurements at baseline appear to be inaccurately high, results are robust and show no difference in ECD status between trial arms. We also found that households across all arms share a notable burden of household environmental risks. Microbial contamination of drinking water does not comply with the Peruvian and WHO standards of zero viable coliforms in potable water samples [49, 50]. Indoor and personal 24-h CO air pollution measurements in our study meet WHO guidelines [51], but kitchen PM_2.5_ levels exceed the threshold of 25 μg/m^3^ recommended by the WHO (213 μg/m^3^) [52].

There is an important need for implementing effective national ECD programmes to address the burden of poor development in early childhood. Experiences demonstrate that implementing ECD programmes in low- and middle-income countries is feasible and cost-effective [53]. Our trial offers an ideal opportunity to generate strong evidence regarding the PNCM programme’s efficacy and support the Peruvian national government in its expansion on a wider scale.

The use of active surveillance in this trial will maximise sensitivity while passive surveillance increases the likelihood that true cases will be diagnosed, increasing specificity [54]. To synchronise ARI diagnosis with new national guidelines (stipulated by the MINSA) and address potential limitations of passive ARI surveillance, we equipped all the health centres in our working area with PPOs. Both, health and project field staff were trained, monitored and supervised monthly in the use of PPOs to assess ARI cases equally. PPOs have been underutilised in resource-limited health care settings for a variety of reasons, including high cost, inadequate supply and lack of training [55]. Our design overcomes these limitations and furthers the research on the usefulness and effectiveness of PPOs for ARI case assessments. In addition, PPOs have been shown to be optimal tools for identifying children with hypoxemia, an indicator for severe respiratory diseases [56]. Previous research indicated that the reference SpO_2_ thresholds for hypoxemia are lower at high altitudes in comparison to those at sea level [57]. A median SpO_2_ hypoxemia threshold of 96% for children below 5 years living at 2500 m.a.s.l has been proposed [58]. Our SpO_2_ results in healthy children living between 2250 and 3900 m.a.s.l are lower than the suggested threshold. Hence, considering specific SpO_2_ hypoxemia thresholds for children living in high altitudes becomes a challenge. Our systematic health data collection using PPOs may further pave the way for assessing SpO_2_ hypoxemia threshold values at high altitude settings for children with and without related ARI symptoms [59].

Little attention has been paid to users’ perspectives and preferences in the design of ICS interventions in the past [60]. We conducted a community consultation to select an ICS model that was both locally accepted and efficient. According to recent evidence, those who participate in community consultations to select ICS models have a higher likelihood of increasing ICS use over time [61].

Our study has some limitations. Because of the nature of the trial design, interventions could not be blinded. The use of tablet-based technologies to measure pulse oximetry was a challenge for both health centre and field staff. We trained the health centre personnel on the correct use and benefits of introducing PPOs in their daily work in two group sessions. However, due to frequent changes of the personnel in health centre, we had to retrain new staff individually on-site on a monthly basis. Baseline ECD evaluation and the age-specific assessment tools were not as straightforward to apply as we had experienced previously [25]. Despite the assertion from the PNCM programme experts that novices could also use the ESDI tool, we found that the lack of familiarity and experience among the field staff consistently produced unusually high scores in all individuals (at baseline). However, in the context of this baseline assessment, the screening was conducted to assess the relative ECD status between study arms as opposed to estimate absolute differences in ECD psychomotor and cognitive indicators. Hence, overall high scores do not invalidate our conclusion that randomisation was successful given that study arms had similar ECD levels at baseline. To diminish any further potential bias at the end of study, we will apply both the ESDI and BSID tools.

Despite the limitations, we believe that the IHIP-2 trial will generate needed evidence on the potential synergistic benefits of combining ECD and environmental health interventions. We actively sought to involve national and governmental actors (i.e. PNCM, SENCICO, MINSA) in developing our interventions and throughout the study to foster sustainability and reinforce collaborative engagements in the future.

## Conclusion

In this paper, we present the baseline results of a factorial cluster-randomised trial evaluating an integrated home-environmental intervention package and an early child development intervention in children under 36 months of age living in rural Andean Peru. The trial arms were balanced with respect to most baseline characteristics, air and water contamination and child’s developmental status. Baseline results determine that the trial’s randomisation was successful and study arms are comparable for analysis at the end of study. The results of this trial will yield valuable information for assessing the synergic, rational and cost-effective benefits of the combination of home-based interventions.

## Supplementary information

**Additional file 1.** Theory of Change: Integrating early child development in home-based environmental interventions in rural Peru. This appendix depicts the theory of change of the study. Within it, we identify several processes and key performance indicator (i.e.: number of household’s enrolled, % of water samples collected, % of ECD assessments, % of anthropometric measurements, among others) that were monitored throughout the study to evaluate the implementation process and increase the effectiveness of the trial.

## Data Availability

The datasets used and/or analysed during the current study are available from the corresponding author on reasonable request.

## Abbreviations

AAF: Acompañamiento a Familias
ARI: Acute respiratory infections
BSID: Bayley Scales of Infant and Toddler Development tool
CBT: Capacity building team
CO: Carbon monoxide
ECD: Early child development
ESDI: Peruvian infant development scale tool
EV: Environmental team
FW: Fieldworker team
HAP: Household air pollution
ICS: Improved cookstoves
IHIP: Integrated home-based intervention package
MINSA: Peruvian Ministry of Health
MF: Mother facilitators
PM_2.5_: fine particulate matter
PNCM: Program Nacional Cuna Mas
PPO: Portable pulse oximeters
PS: Passive surveillance team
SENCICO: Peruvian national industrial certification authority
SpO_2_: Oxygen saturation in blood
TA: Technical assistant team
WHO: World Health Organization

## References

[1] Lakshmi A, Jamal S (2012). Assessing vulnerability of women to indoor air pollution. Res J Environ Earth Scie.

[2] Global Burden of Disease Study 2017 Risk Factor Collaborators (2018). Global, regional, and national comparative risk assessment of 84 behavioural, environmental and occupational, and metabolic risks or clusters of risks for 195 countries and territories, 1990–2017: a systematic analysis for the Global Burden of Disease Study 2017. Lancet.

[3] Bonjour S, Adair-Rohani H, Wolf J, Bruce NG, Mehta S, Prüss-Ustün A (2013). Solid fuel use for household cooking: country and regional estimates for 1980-2010. Environ Health Perspect.

[4] World Health Organization, United Nations Children’s Funds. Progress on Drinking Water and Sanitation. Geneva: World Health Organization, UNICEF; 2014.

[5] Smith KR, Bruce N, Balakrishnan K, Adair-Rohani H, Balmes J, Chafe Z (2014). Millions dead: how do we know and what does it mean? Methods used in the comparative risk assessment of household air pollution. Annu Rev Public Health.

[6] Lim SS, Vos T, Flaxman AD, Danaei G, Shibuya K, Adair-Rohani H (2012). A comparative risk assessment of burden of disease and injury attributable to 67 risk factors and risk factor clusters in 21 regions, 1990-2010: a systematic analysis for the global burden of disease study 2010. Lancet..

[7] Darvesh N, Das JK, Vaivada T, Gaffey MF, Rasanathan K, Bhutta ZA (2017). Water, sanitation and hygiene interventions for acute childhood diarrhea: a systematic review to provide estimates for the lives saved tool. BMC Public Health.

[8] Cohen A, Colford JM (2017). Effects of boiling drinking water on diarrhea and pathogen-specific infections in low- and middle-income countries: a systematic review and meta-analysis. Am J Trop Med Hyg.

[9] Balmes JR, Eisen EA (2018). Household Air Pollution and Chronic Obstructive Pulmonary Disease. “A Riddle, Wrapped in a Mystery, Inside an Enigma”. Am J Respir Crit Care Med.

[10] Balmes JR (2019). Household air pollution from domestic combustion of solid fuels and health. J Allergy Clin Immunol.

[11] Dangour AD, Watson L, Cumming O, Boisson S, Che Y, Velleman Y (2013). Interventions to improve water quality and supply, sanitation and hygiene practices, and their effects on the nutritional status of children. Cochrane Database Syst Rev.

[12] Prüss-Ustün A, Wolf J, Bartram J, Clasen T, Cumming O, Freeman MC (2019). Burden of disease from inadequate water, sanitation and hygiene for selected adverse health outcomes: an updated analysis with a focus on low- and middle-income countries. Int J Hyg Environ Health.

[13] World Health Organization. Mortality from household air pollution. Global Health Observatory (GHO) data, World Health Organization; 2016. Available from: https://www.who.int/gho/phe/indoor_air_pollution/burden/en/.

[14] Grantham-McGregor S, Cheung YB, Cueto S, Glewwe P, Richter L, Strupp B (2007). Developmental potential in the first 5 years for children in developing countries. Lancet..

[15] Walker SP, Wachs TD, Grantham-McGregor S, Black MM, Nelson CA, Huffman SL (2011). Inequality in early childhood: risk and protective factors for early child development. Lancet..

[16] Ngure FM, Reid BM, Humphrey JH, Mbuya MN, Pelto G, Stoltzfus RJ (2014). Water, sanitation, and hygiene (WASH), environmental enteropathy, nutrition, and early child development: making the links. Ann N Y Acad Sci.

[17] Lanphear BP (2015). The impact of toxins on the developing brain. Annu Rev Public Health.

[18] Siddiqi A, Irwin L, Hertzman C. Early child development : a powerful equalizer: final report for the World Health Organization’s Commission on the Social Determinants of Health. Vancouver; 2007. (Human Early Learning Partnership & Commission on Social Determinants of Health).

[19] Maggi S, Irwin LJ, Siddiqi A, Hertzman C (2010). The social determinants of early child development: an overview. J Paediatr Child Health.

[20] Wolf J, Hunter PR, Freeman MC, Cumming O, Clasen T, Bartram J (2018). Impact of drinking water, sanitation and handwashing with soap on childhood diarrhoeal disease: updated meta-analysis and meta-regression. Tropical Med Int Health.

[21] Hartinger SM, Commodore AA, Hattendorf J, Lanata CF, Gil AI, Verastegui H (2013). Chimney stoves modestly improved indoor air quality measurements compared with traditional open fire stoves: results from a small-scale intervention study in rural Peru. Indoor Air.

[22] Thomas E, Wickramasinghe K, Mendis S, Roberts N, Foster C (2015). Improved stove interventions to reduce household air pollution in low and middle income countries: a descriptive systematic review. BMC Public Health.

[23] Smith KR, McCracken JP, Weber MW, Hubbard A, Jenny A, Thompson LM (2011). Effect of reduction in household air pollution on childhood pneumonia in Guatemala (RESPIRE): a randomised controlled trial. Lancet..

[24] Petrovic O, Yousafzai A (2013). Promoting Care for Child Development in community health services, a summary of the Pakistan early child development scale-up (PEDS) trial. Main findings, delivery strengths and the path forward.

[25] Hartinger SM, Lanata CF, Hattendorf J, Wolf J, Gil AI, Obando MO (2017). Impact of a child stimulation intervention on early child development in rural Peru: a cluster randomised trial using a reciprocal control design. J Epidemiol Community Health.

[26] Landrigan PJ, Fuller R, Acosta NJR, Adeyi O, Arnold R, Basu NN (2018). The Lancet Commission on pollution and health. Lancet.

[27] Vazir S, Engle P, Balakrishna N, Griffiths PL, Johnson SL, Creed-Kanashiro H (2013). Cluster-randomized trial on complementary and responsive feeding education to caregivers found improved dietary intake, growth and development among rural Indian toddlers. Matern Child Nutr.

[28] Hartinger SM, Lanata CF, Hattendorf J, Gil AI, Verastegui H, Ochoa T (2011). A community randomised controlled trial evaluating a home-based environmental intervention package of improved stoves, solar water disinfection and kitchen sinks in rural Peru: rationale, trial design and baseline findings. Contemp Clin Trials.

[29] Hartinger SM, Lanata CF, Hattendorf J, Verastegui H, Gil AI, Wolf J, et al. Improving household air, drinking water and hygiene in rural Peru: a community-randomized-controlled trial of an integrated environmental home-based intervention package to improve child health. Int J Epidemiol. 2016;45(6):2089–99. 10.1093/ije/dyw242 PMC5841839 27818376

[30] Clasen T, Smith KR (2019). Let the “a” in WASH stand for air: integrating research and interventions to improve household air pollution (HAP) and water, sanitation and hygiene (WaSH) in low-income settings. Environ Health Perspect.

[31] Singla DR, Kumbakumba E, Aboud FE (2015). Effects of a parenting intervention to address maternal psychological wellbeing and child development and growth in rural Uganda: a community-based, cluster randomised trial. Lancet Glob Health.

[32] Aboud FE, Singla DR, Nahil MI, Borisova I (2013). Effectiveness of a parenting program in Bangladesh to address early childhood health, growth and development. Soc Sci Med.

[33] Resultados Definitivos de los Censos Nacionales 2017 – Censos Nacionales 2017 [Internet]. [cited 2019 Mar 6]. Available from: http://censo2017.inei.gob.pe/resultados-definitivos-de-los-censos-nacionales-2017/.

[34] Hayes RJ, Bennett S (1999). Simple sample size calculation for cluster-randomized trials. Int J Epidemiol.

[35] Moulton LH (2004). Covariate-based constrained randomization of group-randomized trials. Clin Trials.

[36] Gil AI, Lanata CF, Hartinger SM, Mäusezahl D, Padilla B, Ochoa TJ (2014). Fecal contamination of food, water, hands, and kitchen utensils at the household level in rural areas of Peru. J Environ Health.

[37] SENCICO (2011). Reglamento para la evaluación y certificación de la cocina mejorada.

[38] Programa Nacional Cuna Mas (2016). Escala de desarrollo infantil. Manual de aplicación.

[39] World Health Organization (2005). The Treatment of Diarrhoea. A manual for physicians and other senior health workers.

[40] World Health Organization. Handbook: IMCI integrated management of childhood. World Health Organization; 2005. https://apps.who.int/iris/handle/10665/42939.

[41] Lanata CF, Rudan I, Boschi-Pinto C, Tomaskovic L, Cherian T, Weber M (2004). Methodological and quality issues in epidemiological studies of acute lower respiratory infections in children in developing countries. Int J Epidemiol.

[42] Petersen CL, Gorges M, Dunsmuir D, Ansermino M, Dumont GA. Experience Report: Functional Programming of mHealth Applications. In: Proceedings of the 18th ACM SIGPLAN International Conference on Functional Programming [Internet]. New York, NY, USA: ACM; 2013 [cited 2019 Aug 14]. p. 357–62. (ICFP ‘13). Available from: http://doi.acm.org/10.1145/2500365.2500615.

[43] Karlen W, Ansermino JM, Dumont G (2012). Adaptive pulse segmentation and artifact detection in photoplethysmography for mobile applications. Conf Proc IEEE Eng Med Biol Soc.

[44] Karlen W, Gan H, Chiu M, Dunsmuir D, Zhou G, Dumont GA (2014). Improving the accuracy and efficiency of respiratory rate measurements in children using mobile devices. PLoS One.

[45] World Health Organization (2006). WHO child growth standards: length/height-for-age, weightforage, weight-for-length, weight-for-height and body mass index-for-age: methods and development.

[46] OXFAM-DELAGUA. OXFAM-DELAGUA Water Testing Kit — Users Manual version 4.2, revised 2009. Marlborough: University of Surrey; 2009.

[47] Bayley N. Bayley Scales of Infant and Toddler Development. (3rd ed.) San Antonio,TX. 3rd ed. San Antonio: The Psychological Corporation.; 2005.

[48] Montgomery AA, Peters TJ, Little P (2003). Design, analysis and presentation of factorial randomised controlled trials. BMC Med Res Methodol.

[49] Ministerio de Salud (2010). Decreto Supremo N° 031–2010-SA- Reglamento de la calidad de Agua para Consumo Humano.

[50] World Health Organization (2004). Guidelines for Drinking Water Quality. 3rd Edition Volume 1 Recommendations.

[51] World Health Organization (2010). WHO guidelines for indoor air quality: selected pollutants.

[52] World Health Organization (2006). Occupational and Environmental Health Team. WHO Air quality guidelines for particulate matter, ozone, nitrogen dioxide and sulfur dioxide : global update 2005 : summary of risk assessment.

[53] Richter LM, Daelmans B, Lombardi J, Heymann J, Boo FL, Behrman JR (2017). Investing in the foundation of sustainable development: pathways to scale up for early childhood development. Lancet.

[54] Hirve S, Singh SP, Kumar N, Banjara MR, Das P, Sundar S (2010). Effectiveness and feasibility of active and passive case detection in the visceral leishmaniasis elimination initiative in India, Bangladesh, and Nepal. Am J Trop Med Hyg..

[55] Spence H, Baker K, Wharton-Smith A, Mucunguzi A, Matata L, Habte T (2017). Childhood pneumonia diagnostics: community health workers’ and national stakeholders’ differing perspectives of new and existing aids. Glob Health Action.

[56] Ginsburg AS, Delarosa J, Brunette W, Levari S, Sundt M, Larson C (2015). mPneumonia: development of an innovative mHealth application for diagnosing and treating childhood pneumonia and other childhood illnesses in low-resource settings. PLoS One.

[57] Schult S, Canelo-Aybar C (2011). Oxygen saturation in healthy children aged 5 to 16 years residing in Huayllay, Peru at 4340 m. High Alt Med Biol.

[58] Rojas-Camayo J, Mejia CR, Callacondo D, Dawson JA, Posso M, Galvan CA (2018). Reference values for oxygen saturation from sea level to the highest human habitation in the Andes in acclimatised persons. Thorax..

[59] Tüshaus L, Moreo M, Zhang J, Hartinger SM, Mäusezahl D, Karlen W (2019). Physiologically driven, altitude-adaptive model for the interpretation of pediatric oxygen saturation at altitudes above 2000 M a.s.l. J Appl Physiol.

[60] Rehfuess EA, Puzzolo E, Stanistreet D, Pope D, Bruce NG (2014). Enablers and barriers to large-scale uptake of improved solid fuel stoves: a systematic review. Environ Health Perspect.

[61] Wolf J, Mäusezahl D, Verastegui H, Hartinger SM. Adoption of clean Cookstoves after improved solid fuel stove Programme exposure: a cross-sectional study in three Peruvian Andean regions. Int J Environ Res Public Health 2017 8;14(7). 10.3390/ijerph14070745. 10.3390/ijerph14070745 PMC5551183 28698468

