# Supplementary material for: A factorial cluster-randomised controlled trial combining home-environmental and early child development interventions to improve child health and development: rationale, trial design and baseline findings
Source: BMC Med Res Methodol. 2020 Apr 2;20:73. doi: 10.1186/s12874-020-00950-y (PMC7115072; doi:10.1186/s12874-020-00950-y)
Supplement: Supplementary file 1 — Additional file 1. Theory of Change: Integrating early child development in home-based environmental interventions in rural Peru. This appendix depicts the theory of change of the study. Within it, we identify several processes and key performance indicator (i.e.: number of household’s enrolled, % of water samples collected, % of ECD assessments, % of anthropometric measurements, among others) that were monitored throughout the study to evaluate the implementation process and increase the effectiveness of the trial. [file 12874_2020_950_MOESM1_ESM.pdf]

Theory of Change: Integrating early child development in home-based environmental intervention in rural Peru.

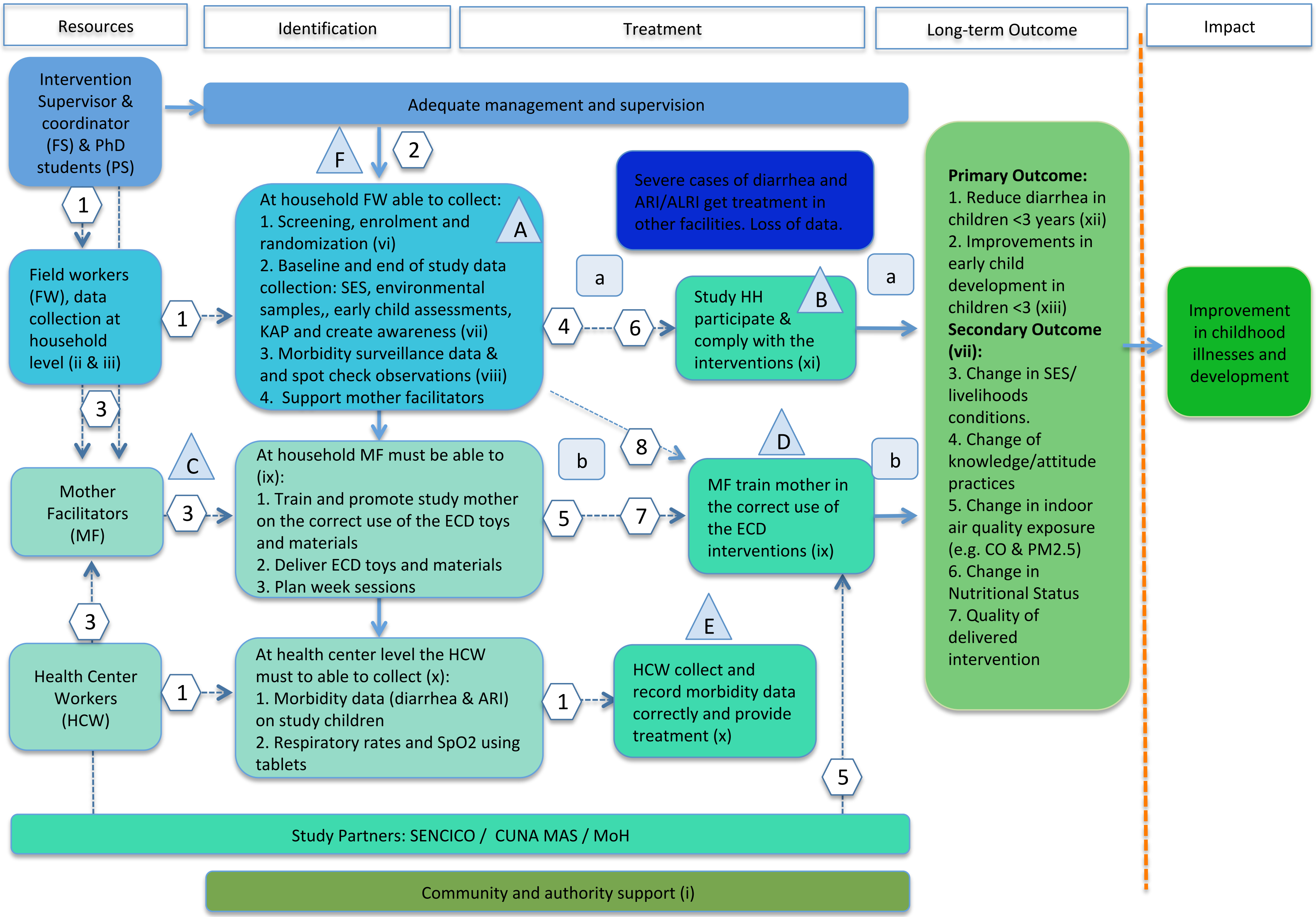

Key

- Intervention Supervision and coordination (FS)
- Intervention Field workers (FW)
- Mother facilitators (MF)
- Health center worker (HCW)
- Institutional Partners (SENCICO)
- Community

- - - - Intervention needed

A Assumption

1 Intervention

a Rationale

(i) Indicator

Assumptions

A. Community families are willing to participate during the duration study and have not plans of moving
B. Study families will comply with all the study requirements (incl. using the improved cook stove exclusively, playing with the child, being available during the weekly for the visits).
C. Sufficient MF available within the communities with the necessary skills, will and time to be trainers.
D. MF are engaged with the study and visit the households weekly for the training with the community mothers. They act proactively and do not criticize the mother or their households.
E. HCW collect the data of the study children when they come for treatment or regular consultations.
F. FS constantly supervise the FW and MF.

Rationale

a. Systematic reviews and meta-analysis evidence show that environmental health interventions that improved access to drinking-water, provided hygiene education or reduced indoor air pollution reduced disease and lead to many other beneficial health and non-health outcomes
b. The Lancet series on ECD mentioned that ECD is crucial for sustainable development and is one of the main social determinants of health i.e. opportunities that are crucial in shaping their lifelong health and development status

Interventions

1. Training of FW and HCW
2. Intervention FS conducts regular supervision of FW and HCW
3. FS and FW recruit MF and conduct training and regular supervision
4. Implementation of interventions: 160 kitchen sink, 160 improved cook stove
5. Implementation of ECD interventions: 160 set of toys (6 deliveries during follow up)
6. Monthly visits for intervention messages reinforcement
7. Weekly household visits for MF training at community houses
8. Monthly group meeting organized by MF and FW at community for group session training

Indicators

i. 100% of the selected communities authorities are aware of the study.
ii. 6 FW trained in data collection tools & identification of signs & symptoms of diarrhea & respiratory illness
iii. 2 Technical assistants (TA) trained in CUNA MAS intervention
iv. 20 MF trained in CUNA MAS interventions
v. 18 HCW are selected to collect morbidity data of study children that go for treatment due to illness, they will be HCW trained in data collection tools & identification of signs & symptoms of diarrhea & respiratory illness
vi. 320 households are enrolled & randomized into 4 study arms.
vii. Baseline & End of study data collection: 100% HH with SES, 100% with water samples collected, 100% HH ECD assessments, 100% anthropometric measurements; subsample of IAQ.
viii. 80% of HH are visited weekly and have complete morbidity surveillance data during follow-up
ix. 80% of HH visits and training session complete during the follow-up period.
x. 90% of morbidity data must be collected at HC from children that went to seek treatment
xi. 90% of household comply with interventions
xii. 20% reduction of diarrhea and 20% reduction of ALRI
xiii. 50% improvement of main ECD indicators (cognitive, language & communication, motor skills)
